# NRIP1 aggravates lung injury caused by *Pseudomonas aeruginosa* in mice by increasing PIAS1 ubiquitination

**DOI:** 10.18632/aging.204027

**Published:** 2022-04-23

**Authors:** Miaoyi Huang, Jianying Li, Jie Bai, Xusheng Du, Hua Guo, Bo Wang, Jiru Xu

**Affiliations:** 1 Department of Microbiology and Immunology, School of Basic Medical Sciences, Xi’an Jiaotong University, Xi’an 710061, China; 2 Department of Respiratory Medicine, Xi'an Central Hospital, Xi’an 710004, China

**Keywords:** NRIP1, UBE2I, PIAS1, lung injury, ubiquitination

## Abstract

Recently, evidence has shown that nuclear receptor interacting protein 1 (NRIP1) is involved in acute lung injury (ALI) progression, but the specific mechanism remains unclear. *Pseudomonas aeruginosa* (PA)-treated TC-1 cells were transfected with pcDNA-NRIP1 or si-NRIP1, and we found that overexpression of NRIP1 inhibited cell viability and promoted cell apoptosis and secretion of inflammatory factors, and transfection of si-NRIP1 reversed these effects. Furthermore, online bioinformatics analysis and co-immunoprecipitation assay results indicated that NRIP1 could bind to Ubiquitin Conjugating Enzyme E2I (UBE2I), and promoted UBE2I expression. Next, the PA-treated TC-1 cells were transfected with si-NRIP1 alone or together with pcDNA-UBE2I, and we observed that transfection with si-NRIP1 inhibited UBE2I expression, promoted cell viability, and reduced cell apoptosis and inflammatory factor secretion, which could be reversed by UBE2I overexpression. Moreover, UBE2I could bind to protein inhibitor of activated signal transducer and activators of transcription 1 (PIAS1). Overexpression of NRIP1 promoted UBE2I expression and inhibited PIAS1 expression, and NRIP1 promoted PIAS1 ubiquitination and degradation by UBE2I. The PA-treated TC-1 cells were transfected with si-UBE2I alone or together with si-PIAS1, and the results indicated that transfection of si-UBE2I had the same effect as transfection of si-NRIP1. Finally, our *in vivo* findings indicated that the expression of NRIP1 and UBE2I was decreased, and PIAS1 expression was increased, in the lung tissues of mice with NRIP1 knocked-down, and the inflammatory infiltration in the lung tissue was reduced. In conclusion, our study demonstrates that NRIP1 aggravates PA-induced lung injury in mice by promoting PIAS1 ubiquitination.

## INTRODUCTION

Acute lung injury (ALI) is a group of clinical syndromes triggered by a variety of factors. In the etiological classification of pneumonia, bacterial pneumonia accounts for approximately 80% of pneumonia incidence [1]. *Pseudomonas aeruginosa* (PA) is a common acquired pathogen, especially important infecting bacteria in diseases such as bronchiectasis, chronic bronchitis, and cystic pulmonary fibrosis. A prospective result suggested a nearly 3-fold increase in the prevalence of PA infection in European intensive care unit patients over an 8.5-year observation period [2]. Another study showed that the nosocomial infection rate of PA in respiratory intensive care units of teaching hospitals in Northwest China was as high as 10.7% from 2013 to 2015 [3].

Nuclear receptor interacting protein 1 (NRIP1), is a newly identified activator of nuclear transcription factor-κB (NF-κB) that upregulates NF-κB-mediated inflammatory factors. In macrophages, NRIP1 can promote the activation of NF-κB and upregulate the expression of genes involved in the inflammatory response [4]. In high glucose treated HK-2 cells, Hyperoside downregulated the protein expression of NRIP1 dose-dependently, and reduced apoptosis and inflammatory response [5]. Another study showed that overexpression of NRIP1 induced proinflammatory cytokine release in cardiomyocytes, which could be reversed by p65 NF-κB inactivation. Furthermore, NRIP1 regulated mitochondrial biogenesis, and played an important role in myocardial inflammation and cardiomyocyte energy metabolism [6]. In lipopolysaccharide (LPS) treated RAW264.7 cells and lung tissue with acute lung injury, NRIP1 expression was elevated, PPARγ expression was reduced, and the secretion of inflammatory factors was increased. NRIP1 knockdown could suppress the production of inflammatory mediators and alleviate acute lung injury by inhibiting DNMT3B mediated methylation of the PPARγ promoter [7]. Given that the activation of the NF-κB signaling pathway could be activated by NRIP1, it is likely that NRIP1 may partake in the pathogenesis of acute lung injury.

Protein post-translational modification is one of the major mechanisms that regulate the biological functions of cellular proteins. There are various types of protein modifications, including phosphorylation, acetylation, methylation, ubiquitination and ubiquitination like. Sumoylation like modification (SUMO) is a type of post-translational modification mediated by a variety of small ubiquitin like modifier molecules, which plays an important role in biological actions, including DNA damage repair, immune response, carcinogenesis, cellular senescence, mainly by regulating protein intracellular localization, protein folding and maintaining genomic stability, and altering target gene expression [8–10]. Ubiquitin Conjugating Enzyme E2I (UBE2I), the only member of the E2 family of ubiquitin conjugating enzymes, is widely expressed in mammalian cells. Although the regulatory role of UBE2I in acute lung injury has been hardly reported so far, Yang et al., referred that overexpressed UBE2I was associated not only with poor prognosis of hepatocellular carcinoma but also with immune infiltration in hepatocytes, and the results of RNA sequencing indicated that UBE2I was involved in diseases such as steatohepatitis, liver fibrosis, and inflammation, suggesting a potential role of UBE2I in inflammation [11].

In this study, we explore the specific mechanism of NRIP1 on TC-1 cells infected with PA and verify the regulatory effect of NRIP1 on ALI caused by PA *in vivo*.

## MATERIALS AND METHODS

### Cell culture and transfection

TC-1 cells were obtained from the Global Bioresource Center ATCC (Rockefeller, MD), cultured in RPMI 1640 medium (Gibco, Rockville, MD) containing 10% fetal calf serum (Sigma, St. Louis, MO) with 5% CO_2_ at 37°C. The ectopic expression plasmids for NRIP1 (pcDNA-NRIP1) and UBE2I (pcDNA-UBE2I) and pcDNA3.1 empty plasmid were constructed by RiboBio Co., Ltd (Guangzhou, China) and transfected by using RiboBio Transfection Kit. Small interfering RNAs of NRIP1 (si-NRIP1), si-UBE2I or si-PIAS1 and scrambled siRNA were synthesized by RiboBio. The oligo sequences applied in this study were as follows: si-NRIP1: CAACTGTGACTAGCCTACGAATGAA; si-UBE2I: GGGCTGTTGCTTATGAGCCTCAGAT; si-PIAS1: GCAACTGATGGAGGATGCAGGTGTA; scrambled siRNA: CCTTGTAAAAATGAAAGCCCGTG.

### Animals

Eight-week-old male C57BL/6 mice were purchased from Guangdong Experimental Animal Center, which are housed at room temperature and ~50% humidity, with light/dark per 12 h and ad libitum. A total of 18 mice randomly divided into control group (injection of the same volume of 0.9% sterile saline solution as the PA011), PA group (intratracheally instilled with 50 μL of PA011) and si-NRIP1 group (PA treated mice were injected intraperitoneally with 20 nM si-NRIP1 once daily), with six mice in each group. All experimental procedures were approved by the Ethics Committee of Xi'an Jiaotong University (NO. XJTULAC-2017-1032).

### PA011 infection

Strain PA011 was a laboratory retained strain. Briefly, PA011 was grown on solid Lysogeny Broth (LB) agar plate for 16 h and colonies were resuspended in sterile saline. Mice were anesthetized with 3% isoflurane and then intratracheally instilled with 50 μL (2 × 10^5^ CFU) of PA011.

### MTT assay

Cell viability was measured by MTT assay. Cells were subgrown in 96-well plates (10^5^ cells/well). 20 μL of prepared MTT solution (5 mg/mL) was incubated every well for 4 h. The formed formazan crystals were dissolved in DMSO (150 μL/well) and assessed with a microplate reader (Bio-Rad) at 490 nm.

### Cell apoptosis

Cell apoptosis was detected with the AnnexinV/PI dual staining apoptosis kit (Sigma) and evaluated with flow cytometry according to the protocols of the kit.

### ELISA

TNF-α and IL-1β concentrations in TC-1 cell supernatant and lung tissue homogenates were detected by ELISA kits (Sigma) following the kits’ protocols.

### Co-immunoprecipitation (co-IP) assay

Cells were washed twice with PBS and lysed by RIPA lysis buffer. The supernatant was collected (centrifugated at 1500 g for 15 min), and the interaction of NRIP1-UBE2I was verified by co-IP with 100 μL of NRIP1/UBE2I antibodies-precoated agarose beads to capture the protein complex, followed by Western blotting.

### Western blotting

Total protein from each sample was extracted with RIPA lysis buffer and separated by 12% SDS-PAGE electrophoresis (15 μg/lane, 130 V for 160 min). The protein was Elec-transferred onto PVDF membranes (100 mA for 100 min) and then blocked in 5% nonfat dry milk at 25°C for 3 h and then incubated with the following primary antibodies: GAPDH antibody (1:1000, Abcam, ab8226), NRIP1 antibody (1:2000, Abcam, ab91476), UBE2I antibody (1:1500, Abcam, ab75854), PIAS1 antibody (1:1000, Abcam, ab109388). Then, the membranes were incubated with proper secondary antibody (1:2000, ab6721) for 1 h (37°C). The bands were visualized by using an ECL kit (Pierce) and were exposed in a gel imaging system, with the enrichment quantified by Image J (Bio-Rad).

### Lung wet/dry weight ratio

Mice were euthanized with 150 mg/kg Pentobarbital, and their lungs were harvested and weighed (wet weigh). Lungs were dried for a constant temperature at 80°C for at least 2 days to get the dry weight.

### Lung function assessment

Lung mechanics were measured using the FlexiVent system and FlexiWare software (SCIREQ, Montreal, Canada) under deep anesthesia with 200 mg/kg Ketamine and 15 mg/kg Xylazine.

### Statistical analysis

All statistical analyses were conducted by SPSS (Chinese version 22.0). Measurements from at least triple repetitions are expressed as mean ± SD and their normality was verified with Shapiro-Wilk test and analyzed by one-way ANOVA (three or more groups) followed by Tukey HSD or *t*-test (two groups). *P* < 0.01 is deemed of significance in this study.

### Availability of data and materials

The datasets used during the present study are available from the corresponding author upon reasonable request.

## RESULTS

### Interference with NRIP1 expression promotes viability and inhibits apoptosis in PA-treated TC-1 cells

**Figure 1:**
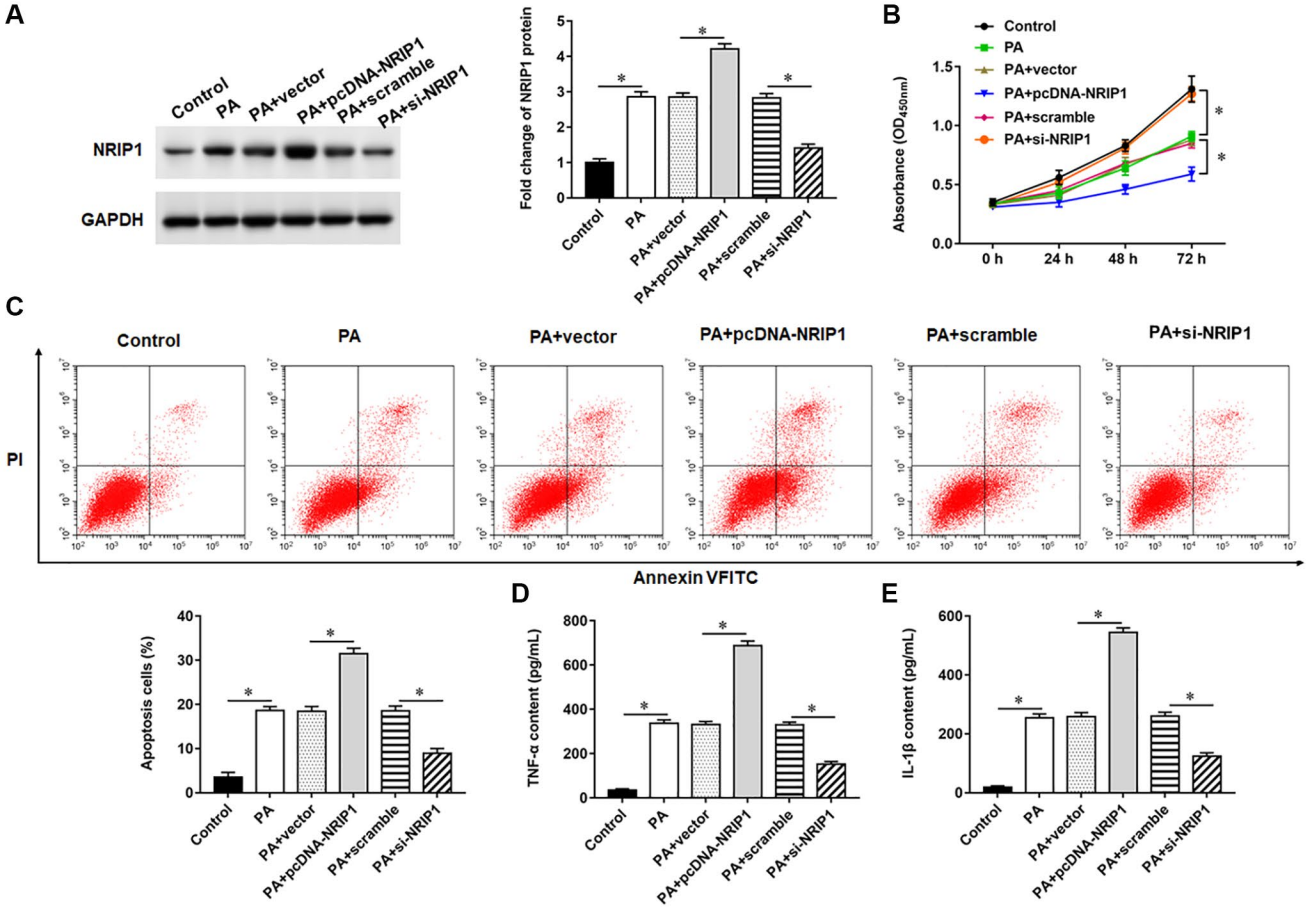
**Effects of NRIP1 on PA treated TC-1 cells. TC-1 cells were treated with PA011, and the pcDNA-NRIP1 or si-NRIP1 was transfected into cells, respectively.** (**A**) The protein expression of NRIP1 was analyzed by Western botting. (**B**) MTT assay was used to analyzed the viability of cells. (**C**) Apoptosis of cells were detected by flow cytometry. (**D**, **E**) Relative content of TNF-α and IL-1β were analyzed by ELISA kits. GAPDH was used as an invariant internal control for calculating protein-fold changes. *N* = 6, ^**^P < 0.01.

TC-1 cells were infected with PA011 alone, or the pcDNA-NRIP1 or si-NRIP1 was transfected into PA011-infected cells for 48 h. NRIP1 protein expression was increased in TC-1 cells infected with PA, and transfection with pcDNA-NRIP1 promoted NRIP1 protein expression, and transfection with si-NRIP1 inhibited NRIP1 expression (Figure 1A). Moreover, PA infection suppressed cell viability and promoted apoptosis and inflammatory responses. Compared with infection with PA alone, transfection with pcDNA-NRIP1 further attenuated cell viability and aggravated cell apoptosis and inflammation, and transfection with si-NRIP1 improved cell viability (Figure 1B) and reduced cell apoptosis (Figure 1C) and inflammation (Figure 1D, 1E).

### NRIP1 positively regulates UBE2I expression

**Figure 2:**
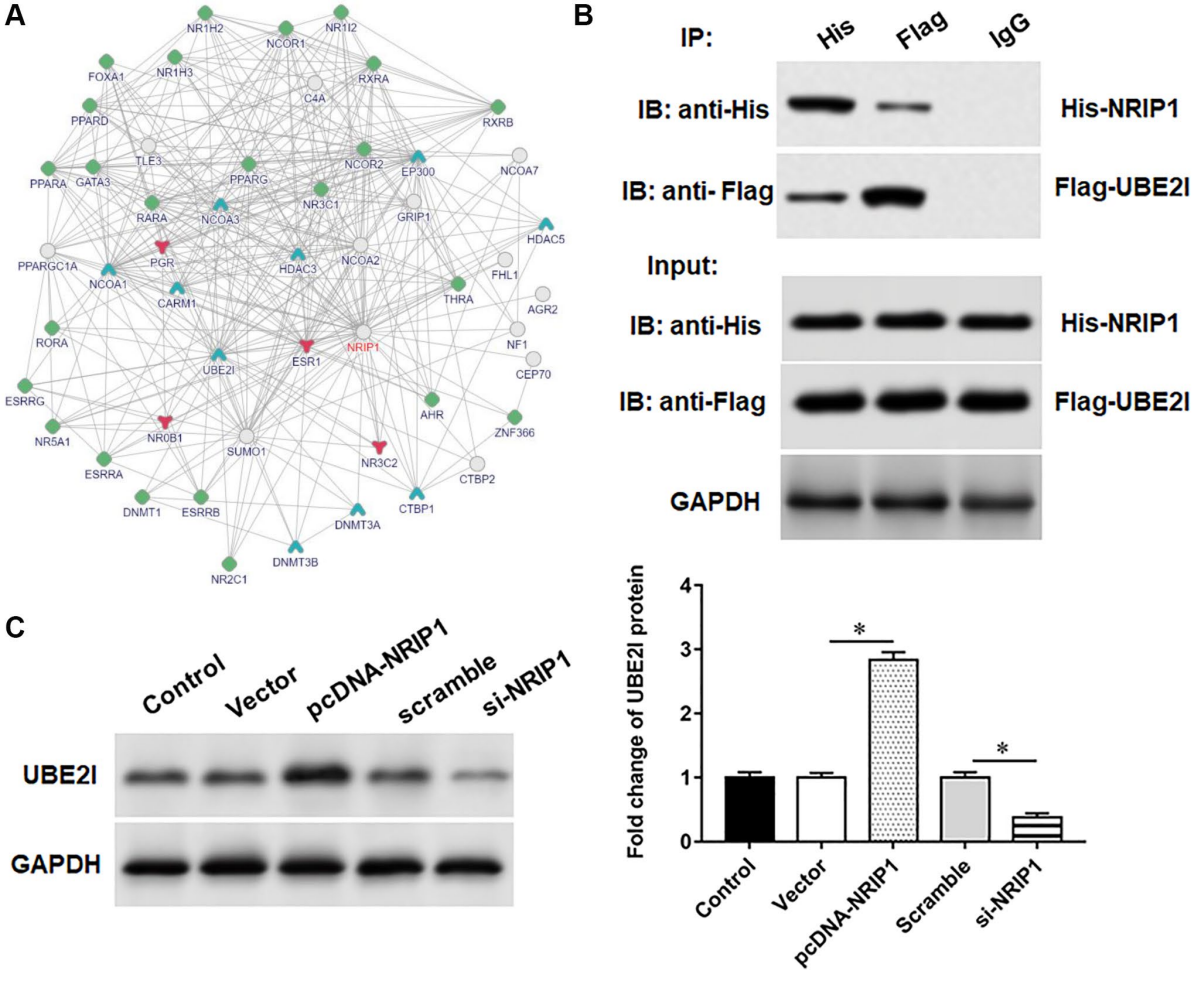
**UBE2I interacts with NRIP1.** (**A**) The online biological database was used to predict the interaction relationship NRIP1 between and UBE2I (https://inbio-discover.intomics.com/map.html). (**B**) The pcDNA-NRIP1 or si-NRIP1 was transfected into TC-1 cells, respectively, and the protein expression of UBE2I was detected by Western blotting. (**C**) The binding relationship of NRIP1 and UBE2I was validated by co-immunoprecipitation assay. GAPDH was used as an internal reference. *N* = 6, ^*^P < 0.01.

Bioinformatics analysis (output from https://www.intomics.com/inbio/map/#home) suggested that UBE2I protein may interact with NRIP1 (Figure 2A). Transfection of pcDNA-NRIP1 promoted the expression of UBE2I protein, transfection of si-NRIP1 inhibited the expression of UBE2I protein (Figure 2B). Next, co-immunoprecipitation assays showed that both NRIP1 and UBE2I proteins were abundant in immunoprecipitants captured by exogenous NRIP1 antibody or exogenous UBE2I antibody, but not by IgG antibody, indicating that NRIP1 can bind to UBE2I protein (Figure 2C).

### Overexpression of UBE2I inhibits viability and promotes apoptosis in PA-treated TC-1 cells

**Figure 3:**
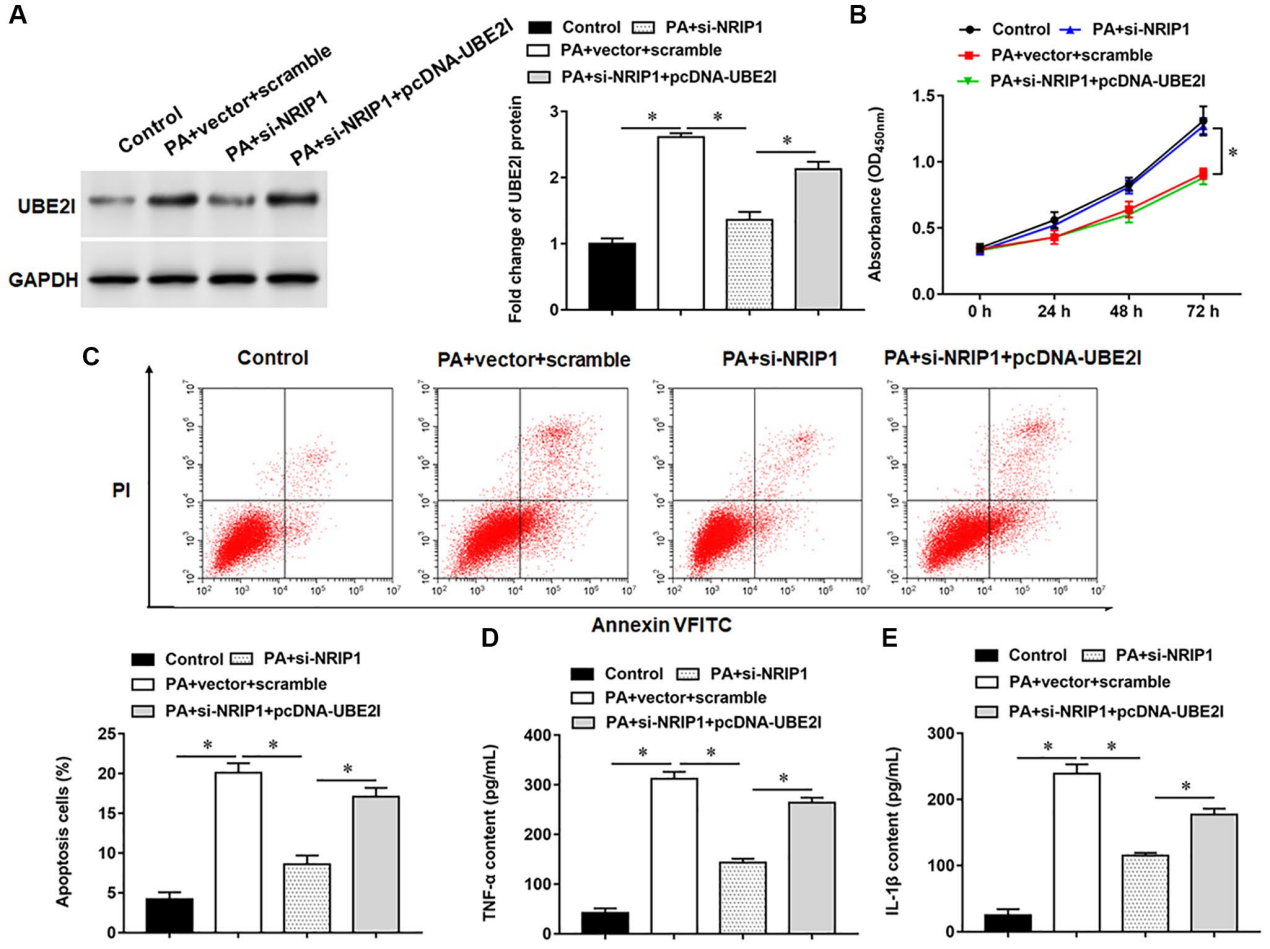
**NRIP1 affects PA treated TC-1 cells by positively regulating UBE2I expression.** The PA011-treated TC-1 cells were transfected with si-NRIP1 alone or together with pcDNA-UBE2I. (**A**) The protein expression of UBE2I was analyzed by Western botting. (**B**) MTT assay was used to analyzed the viability of cells. (**C**) Apoptosis of cells were detected by flow cytometry. (**D**, **E**) Relative contents of TNF-α and IL-1β were analyzed by ELISA kits. GAPDH was used as an internal reference. *N* = 6, ^*^P < 0.01.

UBE2I protein was upregulated in TC-1 cells infected with PA, si-NRIP1 inhibited UBE2I protein expression, and overexpression of UBE2I reversed the effect of si-NRIP1 on UBE2I (Figure 3A). Furthermore, PA infection suppressed cell viability and set off apoptosis and inflammatory responses. Transfection of si-NRIP1 improved cell viability (Figure 3B) and reduced apoptosis (Figure 3C) and inflammation (Figure 3D, 3E), and overexpression of UBE2I reversed the effects of si-NRIP1 in PA-infected cells.

### UBE2I negatively regulates PIAS1 expression

**Figure 4:**
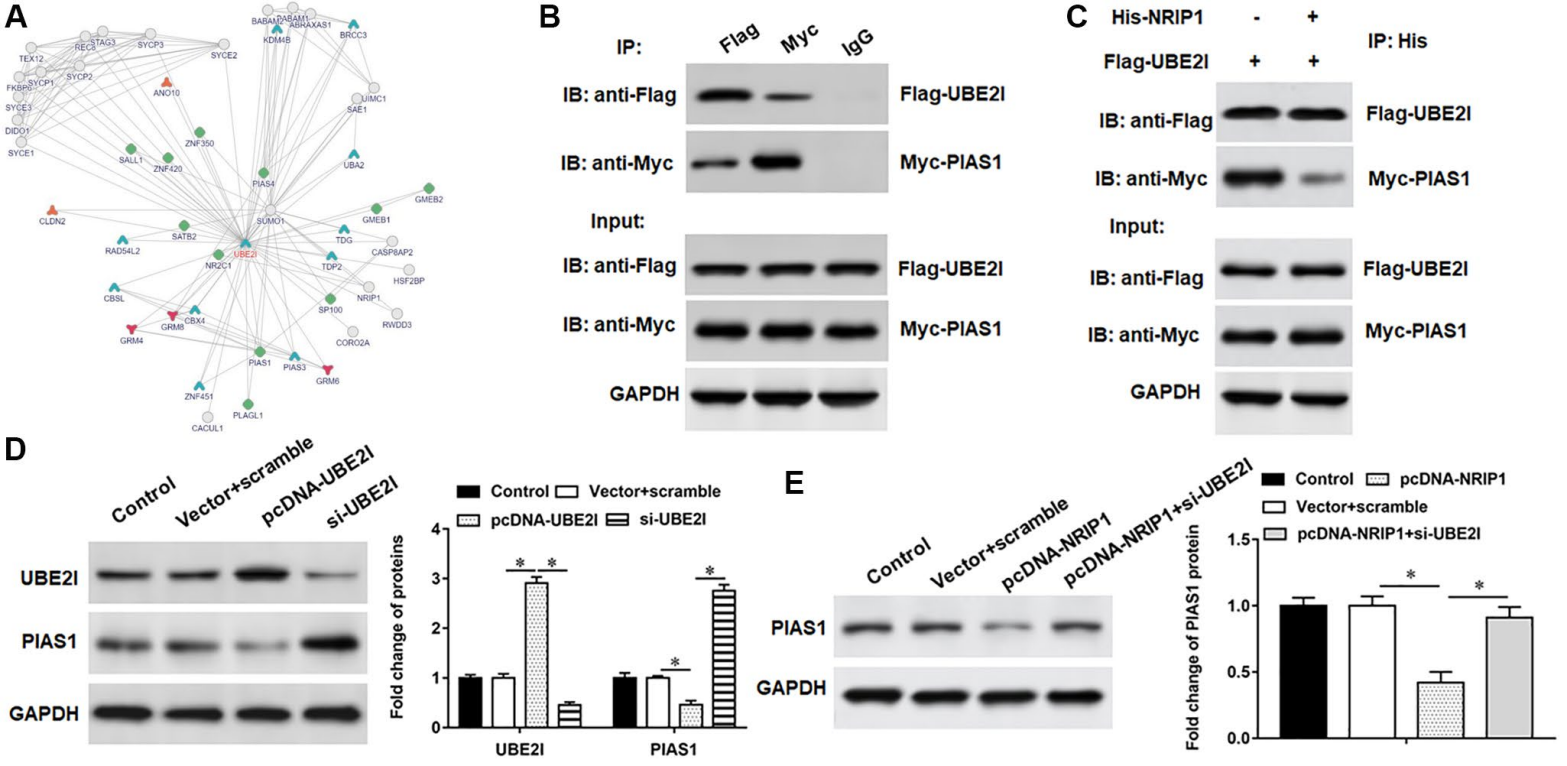
**PIAS1 interacts with UBE2I.** (**A**) The online biological database was used to predict the interaction relationship UBE2I between and PIAS1 (https://inbio-discover.intomics.com/map.html). (**B**) The binding relationship of UBE2I and PIAS1 was validated by co-immunoprecipitation assay. (**C**) Co-immunoprecipitation assay was used to validate the binding relationship of NRIP1, UBE2I and PIAS1. (**D**) The pcDNA-UBE2I or si-UBE2I was transfected into cells, and the protein expression of UBE2I and PIAS1was analyzed by Western botting. (**E**) The cells were transfected with pcDNA-NRIP1 alone or together with si-UBE2I, and the protein expression of PIAS1was analyzed by Western botting. GAPDH was used as an internal reference. *N* = 6, ^*^P < 0.01.

PIAS1 protein may interact with UBE2I (Figure 4A). Next, co-immunoprecipitation demonstrated UBE2I and PIAS1 proteins were abundant in immunoprecipitants captured by exogenous UBE2I antibody or exogenous PIAS1 antibody, but not by IgG antibody, indicating that UBE2I can bind to PIAS1protein (Figure 4B). Furthermore, we observed that overexpression of NRIP1 inhibited PIAS1 protein expression (Figure 4C). The pcDNA-UBE2I promoted UBE2I protein expression and inhibited PIAS1 protein expression, while si-UBE2I transfection had the opposite effect (Figure 4D). Furthermore, NRIP1 negatively regulated PIAS1 expression, and si-UBE2I reversed the its inhibition on PIAS1 expression (Figure 4E).

### UBE2I promotes PIAS1 ubiquitination

**Figure 5:**
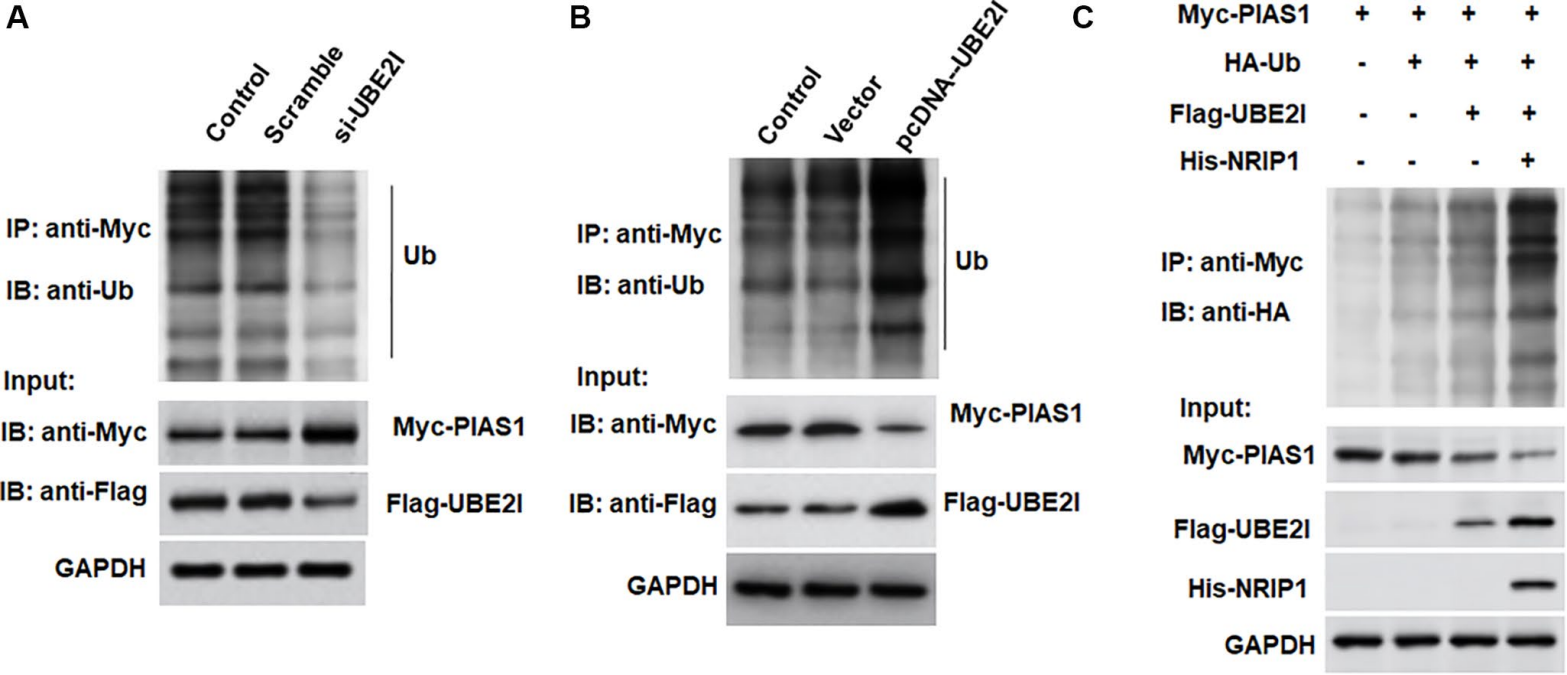
**UBE2I accelerates PIAS1 ubiquitination for degradation.** (**A**) The si-UBE2I was transfected into cells and the ubiquitination level of PIAS1 was detected by co-immunoprecipitation assay. (**B**) The pcDNA-UBE2I was transfected into cells and the ubiquitination level of PIAS1 was detected by co-immunoprecipitation assay. (**C**) UBE2I was overexpressed alone or simultaneously with NRIP1, and the ubiquitination level of PIAS1 was detected by co-immunoprecipitation assay. GAPDH was used as an internal reference. *N* = 6, ^*^P < 0.01.

The pcDNA-UBE2I was transfected into TC-1 cells and immunoprecipitated with an exogenous PIAS1 antibody, followed by immunoblotting with an exogenous ubiquitin antibody, an exogenous PIAS1 antibody, and an exogenous UBE2I antibody, respectively. We observed increased migration of ubiquitinated bands, decreased PIAS1 protein expression, and increased UBE2I protein expression in cells overexpressing UBE2I (Figure 5A). In contrast to overexpression of UBE2I, the ubiquitinated band was decreased, PIAS1 protein expression was increased, and UBE2I protein expression was decreased in cells transfected with si-UBE2I (Figure 5B). Furthermore, we observed that UBE2I promoted the ubiquitination of PIAS1, and NRIP1 enhanced the effect of UBE2I on PIAS1 ubiquitination (Figure 5C).

### Interference with PIAS1 expression inhibits viability and promotes apoptosis in PA-treated TC-1 cells

**Figure 6:**
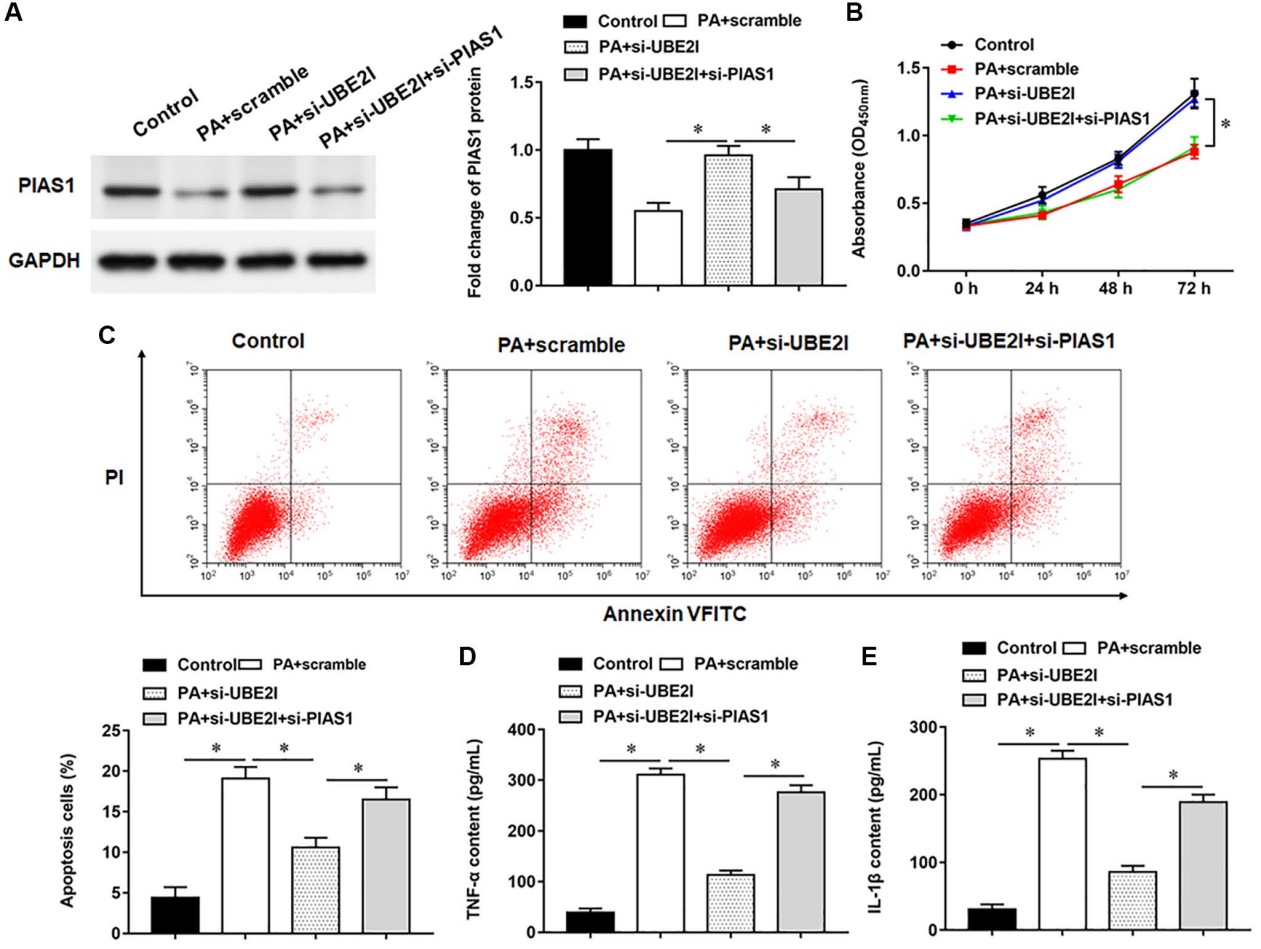
**UBE2I affects TC-1 cells by negatively regulating PIAS1.** The PA011-treated TC-1 cells were transfected with si-UBE2I alone or together with si-PIAS1. (**A**) The protein expression of PIAS1 was analyzed by Western botting. (**B**) MTT assay was used to analyzed the viability of cells. (**C**) Apoptosis of cells were detected by flow cytometry. (**D**, **E**) Relative content of TNF-α and IL-1β were analyzed by ELISA kits. GAPDH was used as an internal reference. *N* = 6, ^*^P < 0.01.

PIAS1 protein was decreased in TC-1 cells infected with PA, and si-UBE2I promoted PIAS1 protein expression, which could be reversed by the transfection with si-PIAS1 (Figure 6A). Furthermore, PA infection suppressed cell viability and set off apoptosis and inflammation. Transfection of si-UBE2I improved cell viability (Figure 6B) and reduced cell apoptosis (Figure 6C) and inflammation in PA-infected TC-1 cells (Figure 6D and 6E), which could be reversed by transfection with si-PIAS1.

### Interference with NRIP1 expression alleviates lung injury in PA infected mice

**Figure 7:**
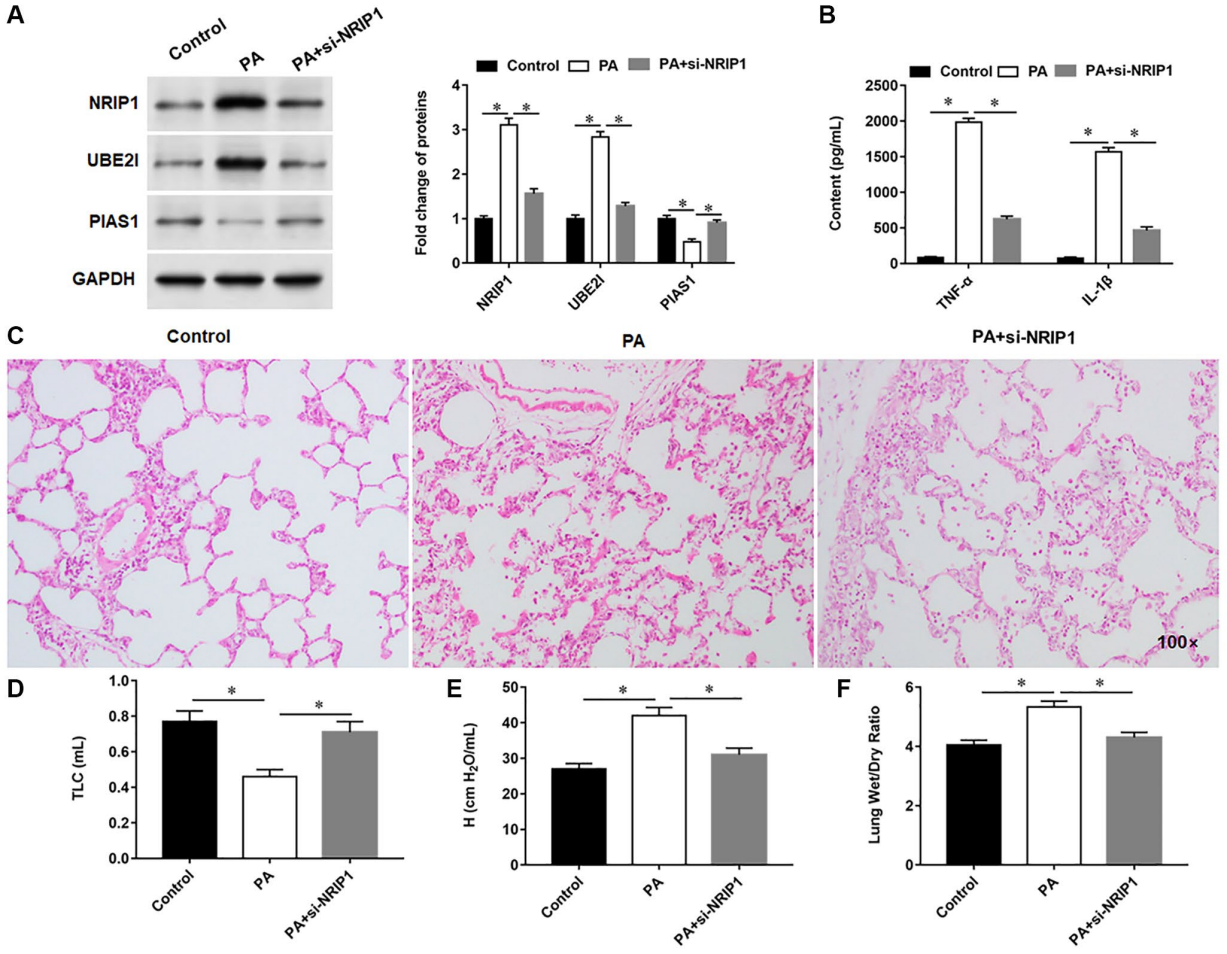
**Interference with NRIP1 expression attenuates lung injury in PA infected mice.** A total of 18 mice randomly divided into control group (injection of the same volume of 0.9% sterile saline solution as the PA011), PA group (intratracheally instilled with 50 μL of PA011) and si-NRIP1 group (PA treated mice were injected intraperitoneally with 20 nM si-NRIP1 once daily), with six mice in each group. (**A**) The protein expression of NRIP1, UBE2I and PIAS1 in lung tissues was analyzed by Western botting. (**B**) Relative content of TNF-α and IL-1β in lung tissues were analyzed by ELISA kits. (**C**) Representative images of HE staining of lung tissues. (**D**, **E**) Total lung capacity (TLC) and tissue elastance (H) were assessed using FlexiWare software. (**F**) The ratio of lung wet-to-dry weight was calculated. GAPDH was used as an internal reference. ^*^P < 0.01.

NRIP1 and UBE2I proteins were upregulated, PIAS1 protein was lowered, and the secretion of inflammatory factors was increased in PA infected lung. Compared with PA infected mice, si-NRIP1-injected mice showed decreased expression of NRIP1 and UBE2I proteins, increased expression of PIAS1 protein (Figure 7A), and decreased secretion of inflammatory factors (Figure 7B) in lung tissues. HE staining results indicated increased inflammatory infiltration and disordered alveolar structure in PA infected lung, which could be improved by si-NRIP1 injection (Figure 7C). Six hours after intratracheal instillation of PA, mice developed typical symptoms of pulmonary edema, manifested by decreased TLC and increased H, and mice in which NRIP1 expression was perturbed had TLC (Figure 7D), attenuated H (Figure 7E), and improved lung function. Similarly, the results of lung wet to dry weight ratio evaluation suggested that PA infection enhanced lung edema, and interference with NRIP1 expression alleviated PA induced lung edema (Figure 7F).

## DISCUSSION

ALI is a life-threatening syndrome manifesting as acute hypoxic respiratory failure, and impairment of lung epithelial function is one of the important mechanisms leading to ALI, which is believed to cause lung epithelial and capillary barrier damage or a leading mechanism of inflammation [12, 13]. Gram negative bacterial infection is a major cause of high morbidity in ALI. Bacterial endotoxin is a potent activator of many inflammatory factors and can mediate the release of a variety of inflammatory mediators from immune cells, triggering the body inflammatory response [14–16]. Therefore, accelerating the epithelial repair process after lung injury may become a potential idea for the treatment of ALI, so it is urgent to discover novel and effective methods to treat this class of diseases.

NRIP1 is located at chromosome 12q11.2, and NRIP1 protein consists of 1158 amino acids with a relative molecular weight size of 140 kDa. One study showed that overexpression of NRIP1 in macrophages leads to macrophage M1 polarization and expansion during inflammatory responses, and conversely, reduced expression of NRIP1 in macrophages had the opposite effect [4]. In addition, the levels of NRIP1, TNF-α and IL-6 were higher in the plasma and peripheral blood mononuclear cells of patients with diabetes and were positively correlated with the index of insulin resistance, suggesting that the increased levels of NRIP1 may be entangled in inflammation and glucose/lipid metabolic disorders in diabetes [17]. In this study, the expression of NRIP1 protein was increased in TC-1 cells and mouse lung tissues infected with PA, which promoted the secretion of TNF-α and IL-6 and aggravated lung epithelial cell injury, demonstrating the proinflammatory role of NRIP1 in inflammatory responses.

Protein inhibitor of activated signal transducer and activators of transcription 1 (PIAS1) was originally reported as a specific inhibitory protein of STAT1 (Signal Transducer And Activator Of Transcription 1). The PIAS1 gene is located on chromosome 15q23 locus and is a member of the PIAS family. Previous studies have confirmed that PIAS1 functions as an anti-inflammatory factor and can inhibit the NF-κB/STAT1 signaling pathway, regulate inflammatory cell adhesion, and inhibit the process of inflammatory injury. Leng et al., showed that miR-483-5p inhibition eased lung injury in septic mice through upregulating PIAS1 protein expression [18]. In addition, another study found that PIAS1 could attenuate the severity of severe acute pancreatitis (SAP) associated with ALI by enhancing anti-inflammatory activity through inhibition of STAT1, which pointed out that rats with SAP injected with PIAS1 adenoviral vector had decreased levels of TNF-α, IL-1β, and IL-6 in serum, attenuated pancreatic tissue damage, decreased activity of the STAT1 pathway, and decreased expression of MMP-9 and ICAM-1 proteins in lung tissue compared with untreated SAP rats [19]. Coon et al., found that PIAS1 was able to be ubiquitinated and degraded by HECTD2, an E3 ubiquitin ligase, thereby increasing the inflammatory response in an experimental pneumonia model, and they developed a small molecule inhibitor, BC-1382, which was able to target HECTD2 to upregulate PIAS1 expression and attenuate LPS and PA induced lung inflammation [20]. PIAS1, an inhibitory protein of STAT6, inhibited IL-4-induced PRMT1 (a downstream gene of NF-κB) expression, which in turn alleviated antigen induced lung inflammation [21]. Consistent with previous studies above, we found that the expression of PIAS1 protein was reduced in PA infected TC-1 cells and lung tissues of mice, resulting in aggravated lung inflammation, further confirming the anti-inflammatory role of PIAS1 in ALI.

UBE2I, that encodes the ubiquitin-conjugating enzyme UBC9, is famous as an oncogene and proinflammatory gene in many types of cancers and non-cancerous tissues [22, 23]. In lung carcinomas, UBC9 was identified as a metastasis-promoting oncogene through enhancing the transcriptional repression activity of the oncogene Slug or suppressing expression of the tumor suppressor gene Sirt1 [24, 25]. However, in non-cancerous lung tissues, the role of UBE2I is hardly revealed. Here, UBE2I was upregulated in PA-infected lung epithelial cells *in vitro* and PA-infected mouse lung *in vivo*, and that UBE2I could bind to the anti-inflammatory regulator PIAS1, promoted its ubiquitination and inhibited its expression, to aggravates PA-induced lung injury and inflammation. What has something to do with our research is that, several years ago, Pereira et al., reported that UBE2I was upregulated in adult worm stages of lung schistosomula [26].

In conclusion, our study found that NRIP1 protein expression was increased in PA infected TC-1 cells and accelerated PIAS1 ubiquitination and degradation by promoting UBE2I protein expression, which in turn promoted TC-1 cell apoptosis and inflammatory responses. Furthermore, the *in vivo* findings confirmed that an increase in NRIP1 protein expression accelerated ALI in PA infected mice, and our findings may provide new insights for treating ALI.
